# Support for smoke-free policy, and awareness of tobacco health effects and use of smoking cessation therapy in a developing country

**DOI:** 10.1186/1471-2458-11-572

**Published:** 2011-07-18

**Authors:** Ellis Owusu-Dabo, Sarah Lewis, Ann McNeill, Anna Gilmore, John Britton

**Affiliations:** 1 UK Centre for Tobacco Control Studies, Division of Epidemiology and Public Health, University of Nottingham, Clinical Sciences Building, City Hospital, NG 5 1PB, UK; 2 Department of Community Health, School of Medical Sciences, College of Health Sciences, Kwame Nkrumah University of Science and Technology, Kumasi, Ghana; 3 School for Health, University of Bath, Bath & London School of Hygiene and Tropical Medicine, University of London, BA2 TAY, UK; 4 Kumasi Centre for Collaborative Research in Tropical Medicine, College of Health Sciences, School of Medical Sciences, KNUST, Kumasi, Ghana

## Abstract

**Background:**

Preventing an epidemic increase in smoking prevalence is a major challenge for developing countries. Ghana, has maintained a low smoking prevalence despite the presence of cigarette manufacturing for many decades. Some of this success may have been contributed by cultural factors and attitudes. We have studied public awareness of health risks, attitudes to smoke-free policy, tobacco advertising/promotion and other factors in a Ghanaian population sample.

**Methods:**

We used two-stage cluster randomized sampling to study household members aged 14 and over in a representative household sample in the Ashanti Region of Ghana.

**Results:**

6258 people, 88% of those eligible, took part in the study. Knowledge of health risks of smoking and passive smoking was high; radio was the main source of such information. Most people work and/or spend time in places where smoking is permitted. There was very strong support (97%) for comprehensive smoke-free legislation, particularly among Christians and Muslims. Despite the advertising ban, a third of respondents (35%), particularly in urban areas, had noticed advertising of tobacco or tobacco products, on the radio (72%) and television (28%). Among smokers, 76% had attempted to quit in the last 6 months, with the main sources of advice being friends and spouses. Use of nicotine replacement therapy was very rare. Low levels of health awareness were seen in females compared with males (Adjusted Odds Ratio (AOR); 0.51, 95% CI 0.39-0.69, p < 0.001). High levels of health awareness was seen among Traditionalists compared with Christians AOR; 2.16 95% CI 0.79-5.94, p < 0.05) and the relatively well educated (AOR; 1.70 95% CI 1.12-2.58, p < 0.05) and those living in rural areas (AOR 1.46 95% CI 1.14-1.87, p = 0.004).

**Conclusion:**

Awareness of health risks and support for smoke-free policy are high in Ghana. Exposure to tobacco advertising or promotion is limited and most smokers have tried to quit. Whether these findings are cause or effect of current low smoking prevalence is uncertain.

## Background

Substantial progress is now being made in reducing the prevalence of tobacco smoking in many developed countries, so tobacco companies are looking increasingly to the developing world for opportunities to develop new growth in tobacco use. Consequently, tobacco consumption is expected to grow most markedly over the next two decades in the developing world [1,2], and mortality from tobacco to almost double from the current 5 million deaths per annum by 2025 [3]. However, experience in some countries indicates that initiation and subsequent epidemic progress of tobacco consumption is neither inevitable nor unavoidable [4,5], and that whereas support for smoke free legislation is not always translated into comprehensive plans, cultural factors are likely as well to influence both uptake, smoke-free policy, awareness on tobacco side effects, and use of smoking cessation [6,7]. It is therefore important to study countries that have avoided large-scale tobacco epidemics, to identify the policies and cultural factors that are likely to have been responsible and to determine to what extent these policies are being enforced.

Ghana is a developing country in Western Africa that has experienced rapid economic growth [8], has had the presence of tobacco manufacturing and marketing activities by British American Tobacco (BAT) for most of the country's history [9]. In spite of this, Ghana has maintained a relatively low prevalence of tobacco smoking in comparison with other African countries, currently around 4% and high among elderly, unemployed, less affluent men of low educational status [10-14]. Although Ghana does not have a written tobacco control policy, it was the first African country to introduce advertising ban in 1982 followed by South Africa in 1998 [9,15]. Smoke-free policy is based on directives from the Minister of Health and covers Ministry of Health buildings and selected premises such as ports, government vehicles and hotels. Other policy initiatives that are of relatively recent origin include, education and information about the health risks of tobacco use and the celebration of *World No Tobacco Day *[16]. We have previously reported that the early imposition of an advertising ban, coupled with political, economic and logistic restraints on manufacturing growth, are likely to have been important in restraining the growth of tobacco use in addition to socio-cultural factors [9,11].

In this paper, we explore the extent to which the public are aware of the health risks of tobacco use, support smoke-free legislation, prevent passive smoke exposure in the home, and to which smokers are advised to, and attempt to quit and whether they have used nicotine replacement therapy based on some socio-demographic characteristics of smokers to further explain the current tobacco use in Ghana.

## Methods

### Study Design

Details of the study methods have been described elsewhere [11]. In brief, we used a two stage cluster randomized sampling design to study a representative sample of all residents aged 14 and over in Ashanti region, in central Ghana. The sampling frame comprised all updated enumeration areas (EA) for the Ashanti Region, each of which typically comprised between 100 and 120 households. We then stratified the list of EAs into equal (15 each) numbers of urban and rural areas and took a random sample of 30 for the study.

Staff members of the Ghana Statistical Service (GSS) of the Ashanti Region then visited each of the selected EAs to identify a systematic sample of 20 households from each. These houses were then visited by trained fieldworkers who explained the purpose of the study, obtained informed consent, and conducted a face-to-face interview of all consenting individuals aged 14 and above using a structured questionnaire. Up to 3 visits were made to contact individuals not present at the first visit. We excluded individuals living in institutions (such as hospitals, prisons and hotels) and foreign nationals. The survey was completed between December 2007 and May 2008.

### Data Collection and Study Variables

Smoking questions were based on those used in the UK General Household Survey [12] and the International Tobacco Control survey (ITC) [17], supplemented by questions on local issues relevant to smoking. Questionnaires were translated and back-translated from English into local Asante Twi language and pilot tested in a sample of 20 households outside the study area. Data on prevalence and uptake of smoking have been reported elsewhere [11]. The questionnaire included a range of questions covering smoking policy at respondent's place of work, support for smoking regulations in indoor public areas, knowledge of health effects of tobacco smoke and tobacco smoke constituents and diseases caused by smoking, attitudes and beliefs about the dangers of different tobacco products, smoking regulation and advertising/promotion of tobacco products, and other factors such as attempts to quit and use of nicotine replacement therapy by smokers.

## Awareness of health promotion campaigns and health risks

In assessing the awareness of health promotion campaigns and health risks, respondents were asked question about advertisements warning about smoking risks, how many of these advertisements they had seen in the previous year. The sources of these adverts were also noted by asking where the advertisements were seen. An assessment was also made about the influence of passive smoking on one's health. In considering the dangers posed to the population by others' smoking, we generated two categories of the responses into either 'agree' or 'disagree', by combining 'strongly agree' and 'agree' into one category and 'neither agree nor disagree', 'disagree' and 'strongly disagree' into a second category for the analysis. Questions were also asked about parental influence on childhood smoking.

## Knowledge of health effects and composition of cigarette smoke

The study assessed the knowledge of the constituents of tobacco smoke by asking the question: "As far as you know, are each of the following chemicals included in cigarettes smoke?" The main constituents assessed were cyanide, mercury, arsenic and carbon monoxide. They were further asked about having noticed about warning labels on cigarette packs in the last six months.

## Awareness and support for smoke-free legislation

Various questions were asked about awareness and support for smoke-free policy in Ghana. To further ask about smoking in places often visited, another question was asked thus; which of the following best describes the rules about smoking in drinking establishments, bars and pubs and answers included smoking not allowed in any indoor areas, smoking is allowed only in some indoor areas and no rules or restriction.

In performing the analysis to ascertain regulations at workplace/school, places often visited and homes, we combined the responses that 'smoking was forbidden in certain areas' and 'smoking forbidden in all areas' into one category, and 'no regulations' into another. In determining whether there was support from the population for preventing smoking in public places, questions were asked about whether people would support law enforcement preventing smoking in public places. In addition to responding about 'public places', respondents were also asked about support for a complete ban or some sort of enforcement. In determining support for smoke free legislation, we combined the responses 'no' and 'don't know' into a single category and 'yes' into another.

## Awareness of tobacco advertising

Questions about tobacco advertising were included. The first part of the question asked respondents about whether they had noticed advertising of cigarettes and/or tobacco products in the last six months, and the source(s) of these advertisements. The second part of the question specifically asked whether the advertisement promoted a tobacco company itself and not just specific brands or products.

## Smoking cessation

Quit attempts by smokers were ascertained by asking whether they had tried to quit smoking in the last 12 months and if so how many times they had done so. Reasons for going back to smoking by smokers were also ascertained. In addition, smokers were asked whether they had been given advice about smoking, and if so, the sources of this advice. Smokers were further asked whether they had heard about medication to help quit smoking, and if so which of the various nicotine replacement therapy formulations (gums, inhalers, patches) or other cessation drugs they had used. Smokers were also asked who had helped them to quit in the previous year.

### Data analysis

Details of data analysis have been published elsewhere [11]. Briefly, for each of the outcome variables stated above, the main exposure variables examined included socio-demographic characteristics such as age, gender, ethnicity, occupation, religion, smoking status and locality type. In determining the relationship of these factors in the multivariate analysis, age, gender and locality type were treated as a priori confounders following a similar process in previous submission [11]. In performing the statistics to ascertain quit attempts among smokers, Fischer's exact test was utilized and we report in table five the corresponding probability values.

In looking at the dangers posed to the population by smoking, we generated two categories of either agree or disagree by combining strongly agree and agree into one and disagree and neither agree nor disagree into another. For analysis of regulations at workplace/school, places often visited and homes, we combined the responses that smoking was forbidden in certain areas and smoking forbidden in all areas into one category, and no regulations into another. Again, in determining support for smoke free legislation, we combined 'no' and 'don't know' into a single variable and yes into another to generate two categorical variables.

Finally, in analyzing for those who had noticed advertising in the past six months we combined 'no' and don't know into a single category and 'yes' into another making them two categories instead of three. Each of these variables generated were then analyzed and tested for levels of significance with various demographic characteristics including age, gender, smoking status, educational background, ethnicity and religion. Univariate and multivariate logistic regression analysis were conducted treating age, gender and locality type as *a priori *confounders using Stata SE version 10 (Statacorp, College Station, Texas, USA). P values less than or equal to 0.05 was accepted to be statistically significant.

### Ethics approvals

Approval for the study was granted by the Committee of Human Research and Ethics of the School of Medical Sciences of the Kwame Nkrumah University of Science and Technology, Kumasi; by the Ethics Review Board of the Ghana Health Service in Accra; and the local ethics committee of the University of Nottingham, UK.

## Results

Details of the socio-demographic characteristics and prevalence have been reported elsewhere [11]. In brief, of the 7096 individuals (2900 male, 4196 females) ascertained to be members of the sampled households and therefore eligible for the study, 6258 (88%; 78% of men and 95% of women) participated. Of these, 2274 (36.3%) were males and 3984 (63.7%) were female, and their median age was 31 (range 14-105). The overall prevalence of current smoking was 3.4% (95% CI 3.0 to 3.9%). 202 (8.9%; 95% Confidence Interval (CI) 7.3 to 10.5%) males and 11 (0.3%; 95% CI 0.1 to 0.4%) females.

### Awareness of health promotion campaigns and health risks

The majority of respondents (84%), and particularly (though not significantly so) non-smokers, and significantly more men (p < 0.001), traditionalists (p < 0.05) and the relatively educated (overall p value p = 0.03, p value for trend pt < 0.05) reported awareness of public health advertisement warnings about the health effects of smoking, typically from the radio or television (Table 1). A higher awareness of such health promotion advertisements was also reported by residents of rural than urban areas (AOR; 1.46, 95% CI 1.14-1.87, p = 0.004). Over half (55%) of all those who recalled health promotion advertisements had noticed them more than ten times. Of those who had noticed health warnings many of them recalled hearing them from the radio (74%), seeing these on television (28%), at the roadside (12%), in the marketplace (4.3%), in newspapers (3.2%), lorry station (2.3%) and other sources (14.4%). There was no association between smoking status and ethnicity in relation to awareness of health promotion advertisements. Nine in ten respondents believed that passive smoking is dangerous to one's health and 94% would object if someone smoked near them, significantly more female than male participants held the latter belief (97%, p value < 0.001) and significantly more older, of those aged 20 years and above, than younger participants (93.6%, p value = 0.02).

**Table 1:** Advertisements warning about tobacco risks, health risks posed by passive smoking and parental influence on children smoking

Characteristic	Number (%)	Number (%) noticed warning	Adjusted odds ratio	Number (%) agree passive smoking dangerous	Adjusted odds ratio	Number (%) agree smoking influences children	Adjusted odds ratio
**Total**	**6258**	5256 (84.0) (7 missing)		5612 (90.0)		5986 (95.7)	
**Age group**							
14-19	1144 (18.3)	740(64.7)	1	1041 (91.0)	1	1094 (95.6)	1
20-29	1686 (26.9)	1605(95.2)	1.21 (0.99-1.48)	1521 (90.2)	1.08(0.79-1.47)	1593 (94.5)	1.49(1.0-2.24)
30-39	1277 (20.4)	1079(84.5)	1.30 (1.07-1.56)	1157 (90.6)	1.07(0.80-1.42)	1233 (96.6)	1.85(1.25-2.75)
40-49	810 (12.9)	710(87.7)	1.68 (1.27-2.22)	721 (88.9)	0.89(0.67-1.17)	787 (97.0)	2.22(1.43-3.45)
50+	1341 (21.4)	1122(83.7)	1.18 (0.89-1.56)	1172 (87.4)	0.78(0.55-1.11)	1279 (95.4)	1.35(1.02-1.79)
		*p = 0.017*		P = 0.056		p = 0.02	
		*Pt = 0.15*		Pt = 0.022		Pt = 0.18	
**Gender**							
Male	2274 (36.3)	2001(88.0)	1	2034 (89.5)	1	2132 (93.8)	1
Female	3984 (63.7)	3255(81.8)	0.60 (0.52-0.70)	3578 (89.8)	1.04(0.81-1.33)	3854 (96.7)	0.51 (0.39-0.69)
		*P < 0.001*		P = 0.74		P < 0.001	
**Locality type**							
Urban	3161 (50.5)	2575(81.6)	1	2845 (90.0)	1	3011 (95.3)	1
Rural	3097 (49.5)	2681(86.7)	1.46 (1.14-1.87)	2767 (89.3)	0.94(0.68-1.29)	2975 (96.1)	1.9 (1.46-2.58)
		*p = 0.004*		P = 0.68		P = 0.30	
**Smoking Status**							
Non smoker	6045 (96.6)	5070(84.0)	1	5437 (90.0)	1	5801 (96.0)	1
Smoker	213 (3.4)	186(87.3)	1.08 (0.66-1.76)	175 (82.2)	0.52(0.31-0.88)	185 (86.9)	0.34(0.20-0.57)
		*p = 0.76*		P = 0.017		P = 0.0002	
**Education**							
Illiterate	1004 (16.0)	810 (80.8)	1	889 (88.6)	1	953 (94.9)	1
Primary	765 (12.2)	644 (84.3)	1.32 (1.05-1.64)	678 (88.6)	0.92(0.65-1.29)	736 (96.2)	1.50 (0.88-2.57)
Secondary	4206 (67.2)	3554 (84.6)	1.42 (1.11-1.81)	3789 (90.1)	1.03(0.77-1.37)	4027 (95.7)	1.50 (0.99-2.28)
Tertiary	283 (4.5)	248 (87.6)	1.70 (1.12-2.58)	256 (90.5)	1.10(0.64-1.92)	270 (95.4)	1.60(0.85-2.99)
		*p = 0.025*		P = 0.90		P = 0.18	
		*Pt < 0.05*		Pt = 0.66		Pt = 0.04	
**Ethnicity**							
Akan	5423 (86.7)	4568 (84.3)	1	4886 (90.1)	1	5193 (95.8)	1
Ewe	59 (0.9)	48 (81.4)	0.84 (0.38-1.87)	54 (91.5)	1.13(0.42-1.29)	57 (96.6)	1.33 (0.35-5.07)
Dagomba	43 (0.7)	39 (90.1)	1.95 (0.54-7.00)	43 (100)	1.23(0.67-1.34)	42 (97.7)	1.74(0.22-3.41)
Other	733 (11.7)	601 (82.2)	0.86 (0.65-1.13)	629 (85.8)	0.65(0.51-0.83)	694 (94.7)	0.79 (0.53-1.18)
		p = 0.575		p = 0.002		p = 0.66	
**Religion**							
Christian	5699(91.1)	4947 (86.8)	1	5107 (89.6)	1	5452 (95.7)	1
Muslim	424 (6.8)	342 (81.0)	0.79 (0.66-0.95)	377 (88.9)	0.94(0.64-1.39)	401 (94.6)	0.85 (0.48-1.51)
Traditionali st	82 (1.3)	77 (93.9)	2.16 (0.79-5.94)	80 (97.6)	5.00 (1.2-20.8)	80 (97.6)	2.24 (0.53-9.42)
Other	53 (0.9)	40 (75.5)	0.47 (0.22-0.98)	48 (90.6)	1.17(0.37-3.73)	53 (100)	2.12 (0.62-3.12)
		P < 0.05		p = 0.190		p = 0.49	

p = Adjusted Wald test; Pt = P value for trend; odds ratio adjusted for age, gender and locality type.

Of the number who believed that passive smoking was dangerous to one's health, 82.6% strongly agreed with the belief that passive smoking was dangerous to one's health, agree (7.1%), neither agree nor disagree (7.8%) and those who disagree or strongly disagree with the belief that passive smoking was dangerous to one's health (2.4%). They were more young (p = 0.06; pt < 0.05), urban than rural (non-significant), non-smokers than smokers (p < 0.05) and the relatively educated particularly those in tertiary education (not significant) holding this belief. The belief that passive smoking was dangerous to one's health was strongly associated with ethnic origin of respondents (p = 0.002). Although respondents had limited knowledge of the harmful constituents of cigarettes as 93% of them did not know anything about the constituents of cigarette smoke (see table 2), there was generally good knowledge about health risks posed by smoking: 97% were aware that smoking causes heart disease, 82% lung cancer, 71% stroke, and 72% mouth and throat cancer.

**Table 2:** Knowledge of health effects and composition of cigarette smoke

Characteristic	Frequency (%)	Male (%)	Female (%)	Age < 20(%)	Age > 20(%)
***Knowledge of diseases caused by smoking***					
Causes heart disease	6055 (96.76)	2187(36.12)	3868(63.38)	882(14.57)	5173(85.43)
Causes stroke	4470 (71.43)	1608(35.97)	2862(64.03)	613(13.71)	3857(86.29)
Causes erectile dysfunction	2796 (44.68)	1017(36.37)	1779(63.62)	365(13.05)	2431(86.95)
Causes lung cancer	5154 (82.36)	1887(36.61)	3267(63.38)	719(13.95)	4435(86.04)
Causes Mouth and throat cancer	4525 (72.31)	1646(36.38)	2879(63.62)	638(14.10)	3887(85.90)
***Knowledge of chemical composition of cigarette smoke***					
Cyanide	56 (0.89)	30(53.57)	26(46.42)	7(12.50)	49(87.50)
Mercury	61 (0.97)	37(65.1)	24(34.9)	15(23.64)	46(76.4)
Arsenic	78 (1.25)	41(57.3)	37(42.7)	7(8.8)	71(91.2)
Carbon monoxide	248 (3.96)	154(66.5)	94(33.5)	148(59.1)	100(40.9)
Don't know	5815 (92.92)	2032(39.4)	3783(60.6)	835(14.36)	4980(85.64)
***Tolerance to smoke by friend***					
Not tolerate	5792(93.57)	1976(88.65)	3816(96.93)	855(95.96)	4937(93.59)
Would tolerate	372(6.4)	252(11.31)	120(3.05)	36(4.04)	336(6.37)

Multiple responses in table

Similarly, awareness of health risks was higher among those aged more than 20 and were predominantly female. Almost 96% of respondents took the view that adult smoking encourages children to smoke. Of these, age (p < 0.05), gender (more women than men, Adjusted Odds Ratio (AOR) 1.97 95% CI 1.49-2.62, p < 0.001) smoking status (non smokers than smokers) and education (a clear trend, the higher the level of education, the more likely they would agree that parental smoking influences children smoking, p = 0.18, pt < 0.05) were associated with the belief that parental smoking influences children in the household to smoke.

### Awareness of and support for smoke-free legislation

Most individuals reported that smoking was permitted in their workplace and/or school and in various public places they often visited (Table 3). Age, gender, locality type, smoking status, education and religion were all associated with awareness of regulation in workplaces or schools. Those working in smoke-free areas tended to be younger people (p < 0.001; pt < 0.001), more educated (the more one's education, the more likely that they were aware of such regulations, adjusted odds ratio of primary education relative to those without any education 1.29, 95% CI 1.05-1.59 Pt < 0.001), non-smokers (smokers were less likely to be aware (AOR; 0.55 95% CI 0.42-0.74), and more likely to live in the urban area (rural dwellers were less likely to have such regulations in places they worked or in school AOR; 95% CI 0.84 0.71-1.00, p < 0.05). Females were less likely to be aware of such regulations in workplaces/schools (AOR; 0.57 95% CI 0.51-0.65, p < 0.001).

**Table 3:** Awareness of and support for smoke-free regulation in Ashanti, Ghana

Characteristic	Regulation at workplace/school	Adjusted odds ratio	Regulation in places often visited	Adjusted odds ratio	Regulation at home	Adjusted odds ratio	Support for smoke free legislation	Adjusted odds ratio
	Number (%) aware of any regulation		Number (%) aware of any regulation		Number (%) aware of any regulation			
**Total**	2349 (37.8)		1924 (30.9)		1715 (27.4)		6904 (97.4)	
	(35 missing)		(24 missing)					
**Age group**								
14-19	570 (63.2)	1	340 (37.6)	1	269 (29.7)	1	885 (97.7)	1
20-29	722 (37.7)	0.36 (0.30-0.44)	612 (31.9)	0.79(0.64-0.96)	501 (26.1)	0.89(0.74-1.07)	1873 (97.4)	0.81 (0.46-1.43)
30-39	416 (32.8)	0.29 (0.24-0.36)	390 (30.7)	0.75(0.60-0.94)	357 (28.0)	0.98(0.77-1.24)	1255 (98.2)	1.19 (0.67-2.11)
40-49	256 (31.7)	0.28 (0.22-0.35)	256 (31.7)	0.79(0.60-1.04)	216 (26.6)	0.90(0.69-1.17)	792 (97.7)	0.87 (0.48-1.60)
50+	385 (28.9)	0.24 (0.19-0.31)	326 (24.5)	0.57(0.45-0.71)	372 (27.7)	0.94(0.75-1.18)	1289 (96.1)	0.48 (0.29-0.80)
	P < 0.001		P < 0.001		P = 0.14		p = 0.004	
	Pt < 0.001		Pt < 0.001		Pt = 0.85		Pt = 0.02	
**Gender**								
Male	1052 (46.5)	1	729 (32.2)	1	721 (31.7)	1	2162 (95.1)	1
Female	1297 (32.8)	0.57 (0.51-0.65)	1195 (30.1)	0.92(0.80-1.05)	994 (25.0)	0.72(0.65-0.80)	3932 (98.7)	4.04 (2.84-5.74)
	P < 0.001		P = 0.20		P < 0.001		P < 0.001	
**Locality type**								
Urban	1251 (39.8)	1	1099 (34.9)	1	815 (25.8)	1	3052 (96.6)	1
Rural	1098 (35.7)	0.84 (0.71-1.00)	825 (26.8)	0.69(0.58-0.83)	900 (29.1)	1.17(0.65-0.80)	3042 (98.2)	2.15 (1.46-3.18)
	P = 0.04		P < 0.001		P = 0.15		p = 0.008	
**Smoking Status**								
Non smoker	2286 (38.0)	1	1871 (31.1)	1	1657 (27.4)	1	5934 (98.2)	1
Smoker	63 (29.6)	0.55 (0.42-0.74)	53 (24.9)	0.73(0.49-1.09)	58 (27.2)	0.81(0.57-1.14)	160 (75.1)	0.08 (0.05-0.14)
	P < 0.001		P = 0.12		P = 0.21		P < 0.001	
**Education**								
Illiterate	286 (28.7)	1	98 (19.9)	1	301 (30.0)	1	980 (97.6)	1
Primary	203 (26.6)	0.69 (0.53-0.90)	199 (26.1)	1.29(1.05-1.59)	209 (27.3)	0.83(0.64-1.07)	744 (97.3)	0.83(0.45-1.53)
Secondary	1689 (40.4)	1.14 (0.53-1.44)	1409 (33.6)	1.76(1.50-2.07)	1110 (26.4)	0.77(0.62-0.96)	4100 (97.5)	1.15 (0.64-2.04)
Tertiary	171 (60.6)	2.63 (1.90-3.65)	118 (41.7)	2.43(1.75-3.37)	95 (33.6)	1.06(0.79-1.43)	270 (95.4)	1.08 (0.49-2.78)
	P < 0.001		P < 0.001		p = 0.031		p = 0.75	
	Pt < 0.001		Pt < 0.001				Pt = 0.53	
**Ethnicity**								
Akan	2075 (38.5)	1	1716 (31.8)	1	1503 (27.7)	1	5277 (97.3)	1
Ewe	25 (42.4)	1.23 (0.58-2.58)	13 (22.0)	0.59(0.27-1.28)	21 (35.6)	1.54(0.79-3.03)	58 (98.3)	1.34 (0.16-11.06)
Dagomba	13 (30.2)	0.79 (0.42-1.47)	6 (14.0)	0.34(0.13-0.87)	16 (37.2)	1.57(0.93-2.65)	41 (95.4)	0.68 (0.18-2.60)
Other	236 (32.5)	0.76 (0.62-0.94)	189 (25.9)	0.74(0.58-0.94)	175 (23.9)	0.81(0.65-1.02)	718 (98.0)	1.35 (0.73-2.49)
	p = 0.06		p = 0.001		p = 0.24		p = 0.68	
**Religion**								
Christian	2156 (38.0)	1	1788 (31.5)	1	1544 (27.1)	1	5562 (97.6)	1
Muslim	137 (32.7)	0.78 (0.61-0.99)	114 (27.0)	0.81(0.61-1.08)	115 (27.1)	0.99(0.79-1.24)	414 (97.6)	1.12 (0.67-1.85)
Trad.	42 (51.2)	1.66 (0.90-3.06)	8 (9.8)	0.25(0.12-0.51)	48 (58.5)	3.20(1.66-6.16)	66 (80.5)	0.15 (0.07-0.32)
Other	14 (26.4)	0.49 (0.23-1.02)	14 (26.4)	0.76(0.45-1.30)	8 (15.1)	0.45(0.20-1.03)	52 (98.1) 4	2.23 (0.41-12.19)
	p = 0.02		p = 0.002		p = 0.002		p = 0.000	

P = Adjusted Wald test; Pt = P value for trend; odds ratio adjusted for age, gender and locality type.

Similarly, about a third of respondents reported having noticed regulations in places often visited and was significantly associated with age (p < 0.001, pt < 0.001), locality type (p < 0.001), education (p < 0.001, pt < 0.001), ethnicity (p = 0.001) and religion (p = 0.002) but did not differ among male and female (p = 0.20). Only 27% of respondents, and more men than women, reported that smoking was prohibited in part or all of their homes. Significantly, of those who were aware of regulations at home more men than women (p < 0.001), educated than none educated (p = 0.03), more in those with tertiary level of education than those with little or no education and religion, in particular those of the Traditionalist faith (p = 0.002). Restrictions on home smoking were not related to education (p = 0.06), urban or rural residence, or smoking status of respondents.

Almost all (97%) respondents were in favour of smoke-free legislation, particularly women (AOR; 4.04 95% CI 2.84-5.74 p < 0.001), rural than urban dwellers (AOR; 2.15 95% CI 1.46-3.18, P < 0.001), the young (p = 0.004, pt = 0.05) and non-smokers as smokers were significantly less likely to support smoke-free policy (AOR; 0.08 95% CI 0.05-0.14, p < 0.001). Support for smoke-free legislation was lower among those of Traditionalist faith (AOR; 0.15 95% CI 0.07-0.32, p = 0.0004) than among Muslims or Christians, but did not differ by education (p = 0.53) and ethnicity (p = 0.68). Most respondents (92%) wanted a complete ban on smoking in a range of public places, including (each of these locations having more than 80% support) churches, mosques, buses, trains, bus stations, waiting areas, airports, shops and bars. Participants cited health consequences, personal dislike of smoking, and economic reasons in support of their opinion.

### Awareness of tobacco advertising

Although a complete ban on advertising was introduced in Ghana in 1982, 35% of participants reported that they had noticed advertising for tobacco products within the past six months (Table 4), and 12% promotion of a tobacco company. Such advertising of tobacco products tended to be seen by smokers, who were 50% more likely to notice them, p = 0.02, by men (p < 0.001), by the more educated (p = 0.07), and substantially more in the urban than the rural areas (p < 0.001).

**Table 4:** Awareness of tobacco advertising and promotion

Characteristic	Noticed tobacco advertising N (%)	Adjusted odds ratio	Noticed promotion for tobacco company N (%)	Adjusted odds ratio
**Total**	**2216 (35.4)**		**767 (12.3)**	
**Age group**				
14-19	279(30.8)	1	108(11.9)	1
20-29	665(34.6)	1.21(1.03-1.42)	238(12.4)	1.04 (0.78-1.37)
30-39	471(36.9)	1.38 (1.15-1.64)	167(13.1)	1.15 (0.86-1.56)
40-49	330(40.7)	1.65 (1.32-2.07)	114(14.1)	1.25 (0.91-1.72)
50+	471(35.1)	1.33 (1.08-1.63)	140(10.4)	0.94 (0.65-0.95)
	*p = 0.003*		*p = 0.09*	
	*Pt = 0.002*		*Pt = 0.52*	
**Gender**				
Male	915 (40.2)	1	314(13.8)	1
Female	1301(32.7)	0.70 (0.60-0.81)	453(11.4)	0.79 (0.66-0.95)
	*P < 0.001*		*p = 0.03*	
**Locality type**				
Urban	1333(42.2)	1	489(15.5)	1
Rural	883(28.5)	0.53 (0.43-0.66)	278(9.0)	0.55 (0.37-0.83)
	*P < 0.001*		*P < 0.001*	
**Smoking Status**				
Non smoker	2108(34.9)	1	734(12.1)	1
Smoker	108 (50.7)	1.50 (1.09-2.07)	33(15.5)	1.13 (0.70-1.84)
	*p = 0.02*		*p = 0.60*	
**Education**				
Illiterate	352 (35.1)	1	89(8.9)	1
Primary	286 (37.4)	1.04 (0.78-1.38)	84(11.0)	1.19 (0.84-1.67)
Secondary	1450 (34.5)	0.80 (0.63-1.02)	548(13.0)	1.34 (0.92-1.95)
Tertiary	128 (45.2)	0.98 (0.67-1.43)	46(16.3)	1.43 (0.99-2.07)
	*p = 0.03*		*p = 0.007*	
	*Pt = 0.07*		*Pt = 0.30*	
**Ethnicity**				
Akan	1904 (35.1)	1	644 (11.9)	1
Ewe	24 (40.7)	1.31 (0.69-2.50)	13 (22.0)	1.95 (0.86-4.40)
Dagomba	30 (69.8)	3.95 (1.97-7.95)	8 (18.6)	1.44 (0.43-4.89)
Other	258 (35.2)	1.00 (0.83-1.21)	102 (13.9)	1.19 (0.91-1.56)
	p = 0.002		p = 0.22	
**Religion**				
Christian	1988 (34.9)	1	685 (12.0)	1
Muslim	150 (35.4)	1.04 (0.85-1.28)	67 (15.8)	1.38 (1.04-1.84)
Traditionalist	57 (69.5)	4.18 (1.81-9.67)	3 (3.7)	0.28 (0.08-0.93)
Other	21 (39.6)	1.04 (0.61-1.77)	12 (22.6)	1.87 (0.87-4.04)
	p = 0.02		p = 0.005	
			Pt = 0.02	

P = Adjusted Wald test; Pt = P value for trend; odds ratio adjusted for age, gender and locality type.

Significantly, age was associated with being aware of such advertisements of tobacco products, the elderly tending to be more aware than those in the younger age groups (overall p = 0.003 and p value for trend pt = 0.002). Awareness differed by ethnic group and was high among Traditionalists (AOR; 4.18 95% CI 1.81-9.67, p = 0.02). Dagombas (the largest tribal grouping in the northern region of Ghana) tended to be more aware of such advertisements about tobacco products (AOR; 3.95 95% CI 1.97-7.95). Awareness of promotion of tobacco companies was significantly associated with gender, locality type, education and religion.

**Figure 1:**
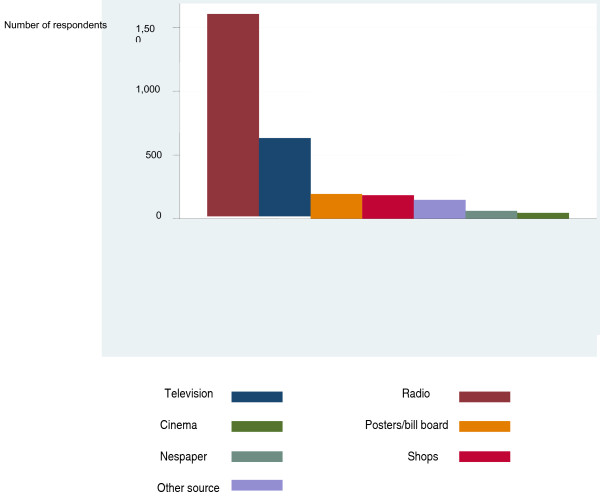
**Sources of tobacco advertisement in Ghana**.

There was significantly less females than males (AOR; 0.79 95% CI 0.66-0.95, p = 0.03) noticing such promotional advertisements by tobacco companies. Significantly less rural, than urban, dwellers noticed such advertisements (AOR; 0.55 95% CI 0.37-0.83, p < 0.001). Although there was a clear trend in the association between promotional advertisements of tobacco companies and education, the overall association was not statistically significant (p = 0.30, pt = 0.007). From Figure 1, the advertising or promotion was most often reported to have been heard on the radio (72%) or seen on the television (28%).

### Smoking cessation

Three quarters (75.9%) of all smokers had tried to quit smoking in the last six months, and the majority of these (69%) had made more than one attempt. The main reasons given for an inability to quit were lack of control of cravings for smoking (57%) and peer influence (30%). Most smokers (76%) had received advice to quit smoking (Table 5), mainly from friends and spouses (65%) and to a much lesser extent from health workers (19%). Just over a third (37%) of all smokers had heard about nicotine replacement therapy (NRT), but only 3 (1.41%) had ever used it (gum). Awareness of NRT was higher in the urban area (X^2 ^= 6.05, p value = 0.02) and in more educated smokers (X^2 ^= 12.66, p value = 0.006). Otherwise there was little difference in smoking cessation behaviour between urban and rural areas or with other socio-demographic factors measured in the study.

**Table 5:** Quit attempts, receiving advice and knowledge of NRT among smokers in Ghana

Characteristic	Current smokers	Quit attempt in last 6 months (%)	Received advice to quit (%)	Heard of NRT (% of current smokers)
**Total**	213 (3.4)	161 (75.9)	160 (75.5)	77 (36.7)
		(1 missing)	(3 missing)	(3 missing)
**Age group**				
14-19	7 (3.3)	3 (42.9)	4 (57.1)	2 (28.6)
20-29	45 (21.1)	36 (81.8)	32 (72.7)	17 (38.6)
30-39	53 (24.9)	43 (81.1)	42 (80.8)	17 (32.7)
40-49	37 (17.4)	29 (78.4)	27 (73.0)	13 (35.1)
50+	71 (33.3)	50 (70.4)	53 (75.7)	28 (40.0)
		*p = 0.15*	*p = 0.64*	*p = 0.91*
**Gender**				
Male	202 (94.8)	154 (76.6)	149 (74.9)	73 (36.7)
Female	11 (5.2)	7 (63.4)	9 (81.8)	4 (36.4)
		*p = 0.30*	*p = 0.60*	*p = 0.98*
**Locality type**				
Urban	113 (53.1)	88 (77.9)	86 (76.8)	50 (44.3)
Rural	100 (47.0)	73 (73.7)	72 (73.5)	27 (27.8)
		*p = 0.52*	*p = 0.63*	*p = 0.02*
**Education**				
Illiterate	29 (13.6)	23 (79.3)	18 (62.1)	9 (31.0)
Primary	36 (16.9)	27 (75)	29 (80.6	19 (52.8)
Secondary	135 (63.4)	100 (84.6)	101 (76.5)	40 (30.3)
Tertiary	13 (6.1)	11 (84.6)	10 (76.9)	9 (69.2)
		*p = 0.91*	*p = 0.34*	*p = 0.006*
**Religion**				
Christian	160 (2.81)	116 (72.96)	120 (75.95)	60 (38.22)
Muslim	18 (4.25)	14 (77.78)	14 (82.35)	4 (22.22)
Traditionalist	29 (35.37)	26 (89.66)	20 (68.97)	9 (31.03)
Other	6 (11.32)	5 (83.33)	4 (66.67)	4 (66.67)
		*p = 0.26*	*p = 0.71*	*p = 0.23*
**Ethnicity**				
Akan	178 (3.28)	137 (76.97)	132 (74.15)	68 (38.42)
Ewe	4 (6.78)	3 (75.00)	3 (75.00)	1(25.00)
Dagomba	4 (9.30)	2 (50.00)	3 (75.00)	1(25.00)
Other	27 (3.68)	20 (74.07)	22 (84.62)	8 (28.57)
		*p = 0.57*	*p = 0.70*	*p = 0.78*

p = Fisher's exact probability test.

## Discussion

This study demonstrates that support for smoke-free policy in work and public places, and awareness of the health hazards of active and passive smoking, are high in the population of a developing country that has to date avoided an epidemic increase in smoking prevalence. It also demonstrates that motivation to quit smoking is also high, though use of cessation support, such as nicotine replacement therapy, is rare. Awareness of and support for smoke-free legislation and awareness of health risks were strongly linked with socio-cultural factors in particular religious faith.

The degree of awareness of current smoke-free policy in Ghana is low as most people reported that smoking was permitted in their workplace/school, places often visited and homes. It has also been demonstrated that although motivation to quit among smokers was high, knowledge and use of medications that help quitting was quite low. The study also shows that the ban on advertising in Ghana, imposed in 1982, has succeeded in preventing the majority of participants from exposure to tobacco advertising, though this is far from absolute.

The study limitations have been discussed in previous publication [11]. They relate to issues of representativeness, recall bias, underrepresentation of males in the sampled population, as well as social coercion in responses given by participants. Given that all tobacco advertising is banned in Ghana it is perhaps surprising that 35% of respondents reported seeing or hearing advertisements. These were reported to be predominantly seen or heard on television and radio, and both these are available from broadcasters based from within and outside Ghana. Those originating from neighbouring countries are not subject to an advertising ban. Anecdotally we are aware that breaches of the advertising ban are also common among small local (FM) radio stations. Awareness of advertising in spite of advertising bans is consistent with findings elsewhere in both developed and developing countries [18,19] and although the reason is uncertain, might be a reflection of poor recall bias among respondents or that adherence to tobacco control measures, in particular advertising bans, is problematic. This perhaps is a reflection of poor tobacco control efforts or inadequacy of the control measures as tobacco control efforts work in a dose response manner, the higher and more comprehensive the ban, the lower the exposure to tobacco marketing [18,9,20]. In South Africa, although tobacco advertising has been banned since 1998, in a study by Reddy et al (2002), 75% of students could recall pro-tobacco advertisements [21]. This therefore perhaps calls for a concerted effort at enforcement of tobacco advertisement bans in these countries including Ghana.

The finding that awareness about smoking regulations in workplaces and schools was high among young people, urban males, non-smokers and the more educated may be a reflection of the likely age of working and schooling class in the sampled population. That smoking regulation was less likely to be noticed by women in the home is surprising as women are traditionally more likely to stay at home and should therefore have the tendency to notice these regulations more than men. The reported levels of support for smoke-free policy in Ghana were extremely high in comparison with (for example) European countries, where a large majority of the population support bans on smoking in workplaces but far fewer, particularly smokers, support such policies in restaurants and bars [7,22].

That support was high among the rural population contrasts with findings from Richmond *et al*, which showed that support for smoke-free legislation was high among urban populations [23]. Support was also particularly high among Christian and Muslim respondents, suggesting that cultural influences and beliefs may be playing a major role in determining specific attitudes toward smoking, and hence to the sustained low prevalence of smoking in Ghana to date [11]. However it is not clear why these factors might have a stronger influence in Ghana than in the many other countries and populations that share these religious beliefs but have become involved in the smoking epidemic.

The observation that there is higher support for smoke free legislation, in particular among women than men, in rural than urban areas and in non-smokers than smokers, is consistent with other reports from developing countries [24-26] and shows that support for smoke free legislation is perhaps higher and stronger in developing countries compared with developed, and that legislation may not necessarily precede support and enforcement for smoke free places in developing countries [27].

The transnational tobacco companies typically try to prevent the enforcement of smoke-free regulations, particularly in developing countries; as for instance in a legal challenge to a strong smoke-free laws passed by the Kenyan parliament [28]. It is also possible that countries in stage I of the smoking epidemic model have a higher tendency to support smoke-free legislation but not enforcement compared with those of stage IV, where passage of laws are very much likely to be adhered to. For example of the population sampled in the Euro barometer survey, two-thirds (73%) were in support of total ban of smoking in offices and other indoor places compared with the over 90% support seen in Ghana.

Implementation of smoke-free legislation remains a challenge in many developing countries including Ghana, Uganda and The Democratic Republic of Congo where partial bans (restrictions) have been implemented without much success [29]. Support in many of these developing countries may not necessarily translate into enforcement and therefore whilst support is a necessary prerequisite for success in the implementation of smoke-free policy it cannot replace enforcement.

The high levels of awareness of health risks associated with smoking in this developing country compares with that pertaining in developed countries [30-32] and perhaps reflects the educational campaigns that the Ministry of Health (MoH) has embarked on in the recent past, in particular during 'World No Tobacco Day' celebrations. This high awareness is unlikely to have arisen from health warnings on cigarette packs as in Ghana, these do not warn about specific disease entities caused by cigarette smoking and currently, health warnings mainly from BAT Ghana Ltd consist of miniscule texts (occupying about 8% max) of cigarette brand packs. The text health warning reads "Ministry of Health Warning; cigarette smoking can be harmful to your health" and consist of Arial narrow font style of size 12 and is therefore unlikely to have contributed to the high level of health awareness among respondents. It is however unclear whether this high level of support and awareness reflect a pre-existing cultural aversion to cigarette smoking, or has arisen from the advertising ban and health promotion policies followed by the Ghanaian government.

That awareness of NRT was higher among educated urban dwellers is not surprising as in many developing countries although there is no extensive provision of tobacco cessation therapies, the few available are likely to be concentrated in urban areas where educational levels are also high and the products are more easily accessible [33,34]. The low levels of awareness of nicotine replacement therapy, and the especially low levels of use of the therapy, does not only illustrate the stage of development of the epidemic [34] but also the need for further health promotion to educate smokers of the effectiveness of cessation support, and for affordable, easily accessible and available formulations.

In many developing countries, there is no legal framework for enforcement of tobacco policies and in cases where there are legal mechanisms they are lax [35]. Our findings suggest that for whatever reason(s), Ghana has succeeded to date in maintaining high levels of support for tobacco control policy, high levels of awareness of health promotion campaigns, and a high willingness on the part of smokers to quit while the 1982 tobacco advertising ban is largely holding. The challenge now is to deliver those policies to prevent future escalation of the smoking epidemic.

## Conclusion

Awareness of health promotion campaigns and health risks is high among the studied population in Ghana. Support for smoke-free policies, are high and so is the willingness to quit smoking in Ghana. The support for smoke-free policy was particularly high among Christians and Muslims, and two thirds of the population is not aware of exposure to tobacco advertising or promotion. Knowledge of constituents of tobacco smoke is low and many smokers are unaware about the use of medications that help with quitting. Whether these high levels of support and of awareness are cause or effect of the current low smoking prevalence in Ghana is still uncertain. Future control policies should emphasize on passage of the national tobacco control bill, implement the ideals of the Framework Convention of Tobacco Control (FCTC), build capacity on tobacco control initiatives and continue to empower the Ghana Health Service and Ministry of Health to continue health education and promotion campaigns on risks of smoking to the general population through the use of pictorial labels for example within the targets set in Conference of Party (CoP) framework. The challenge for Ghana is to implement and sustain these tobacco control efforts to prevent the current situation from escalating particularly targeting populations with specific needs as seen in this study.

## Competing interests

The authors declare that they have no competing interests.

## Authors' contributions

EOD, designed the study, performed data collection, data analysis and drafted the manuscript. SL, AM, AG and JB performed the study design and reviewed the manuscript. All authors read and approved the final manuscript.

## Pre-publication history

The pre-publication history for this paper can be accessed here:

http://www.biomedcentral.com/1471-2458/11/572/prepub
